# Mineralocorticoid Receptors in the Pathophysiology of Vascular Inflammation and Atherosclerosis

**DOI:** 10.3389/fendo.2015.00153

**Published:** 2015-09-28

**Authors:** Mary E. Moss, Iris Z. Jaffe

**Affiliations:** ^1^Tufts Medical Center, Molecular Cardiology Research Institute, Boston, MA, USA; ^2^Sackler School of Graduate Biomedical Sciences, Tufts University School of Medicine, Boston, MA, USA

**Keywords:** mineralocorticoid receptors, aldosterone, atherosclerosis/CAD, vascular inflammation, mineralocorticoid receptor antagonists

## Abstract

Atherosclerosis is a chronic inflammatory disease of the vasculature that causes significant morbidity and mortality from myocardial infarction, stroke, and peripheral vascular disease. Landmark clinical trials revealed that mineralocorticoid receptor (MR) antagonists improve outcomes in cardiovascular patients. Conversely, enhanced MR activation by the hormone aldosterone is associated with increased risk of MI, stroke, and cardiovascular death. This review summarizes recent advances in our understanding of the role of aldosterone and the MR in the pathogenesis of vascular inflammation and atherosclerosis as it proceeds from risk factor-induced endothelial dysfunction and inflammation to plaque formation, progression, and ultimately rupture with thrombosis, the cause of acute ischemia. The role of the MR in converting cardiac risk factors into endothelial dysfunction, in enhancing leukocyte adhesion and infiltration into the vasculature, in promoting systemic inflammation and vascular oxidative stress, and in plaque destabilization and thrombosis are discussed. A greater understanding of the mechanisms by which the MR promotes atherosclerosis has substantial potential to identify novel treatment targets to improve cardiovascular health and decrease mortality.

## Introduction

### Pathophysiology of atherosclerosis

Atherosclerosis is a vascular inflammatory condition that is the primary cause of myocardial infarction (MI), stroke, and limb ischemia. Through substantial research efforts, what was originally thought of as simply a buildup of cholesterol in the arteries is now known to be a chronic inflammatory process involving multiple interacting cell types [reviewed in Ref. (1, 2)]. Briefly, atherosclerosis is triggered by risk factors, such as hyperlipidemia, diabetes, and hypertension, which contribute to injury of the inner endothelial layer of blood vessels. Damage to the endothelium promotes endothelial expression of adhesion molecules, which mediate leukocyte adhesion and migration through the activated endothelium into the underlying tissue. Once in the sub-endothelial space, activated macrophages engulf lipid particles, become foam cells, and release cytokines that attract additional leukocytes. Vascular smooth muscle cells (SMCs) also become activated, in part by factors released from plaque leukocytes, and migrate into the plaque and contribute to a fibrotic cap covering the highly thrombogenic core of lipids and inflammatory cells. This cascade of events forms the mature atherosclerotic plaque. Inflammatory cells and plaque SMCs secrete matrix metalloproteases (MMPs), which destabilize the fibrous cap and predispose the plaque to rupture. Ruptured plaques induce thrombus formation that occludes blood flow to cardiac tissue causing MI, to the brain causing stroke, or to the peripheral vasculature causing limb ischemia.

### Mineralocorticoid receptors and atherosclerosis

The mineralocorticoid receptor (MR) is well-known as a regulator of blood pressure in the kidney, where it promotes sodium retention when activated by its hormone ligand aldosterone (Aldo). MR can also be activated by other mechanisms, including glucocorticoids in tissues lacking the cortisol-inactivating enzyme 11beta-hydroxysteroid dehydrogenase-2 and ligand-independent activation by mechanisms still under investigation. MR-independent effects of Aldo have also been reported. These additional mechanisms will not be discussed as they have been reviewed elsewhere [reviewed in Ref. (3–6)] and are beyond the scope of this review that focuses specifically on the role of non-renal MR in the pathogenesis of atherosclerosis. In addition to its effects on renal sodium handling, clinical studies suggest a more direct role for the MR in cardiovascular pathology in humans. In the RALES, EPHESUS, and EMPHASIS heart failure trials, MR antagonists decreased cardiovascular morbidity and mortality and prolonged survival when compared with placebo controls (7, 8). The observed benefits of MR inhibition were out of proportion to modest decreases in blood pressure, suggesting that MR blockade may exert beneficial cardiovascular effects in tissues outside the kidney. Indeed, Milliez et al. (9) demonstrated that individuals with abnormal MR activation due to hyperaldosteronism experienced stroke, MI, and atrial fibrillation at 4, 6, and 12 times the rate of blood pressure-matched controls. Higher plasma Aldo, even within the normal range, is associated with a significantly increased risk of MI, stroke, and cardiovascular death in patients with coronary artery disease (10) and is an independent risk factor for the progression of carotid atherosclerotic plaques (11). These data suggest an extra-renal role for the MR in the pathophysiology of atherosclerosis that may contribute substantially to cardiovascular morbidity and mortality.

Pre-clinical data in animal models also support a role for extra-renal MRs in atherogenesis. The apolipoprotein E knockout (ApoE^−/−^) mouse model rapidly develops atherosclerotic plaques that mimic the human pathology. Using this model, Tikellis et al. (12) investigated effects of renin–angiotensin–aldosterone system (RAAS) activation by low-sodium intake on the development of atherosclerosis. Low-sodium intake accelerated atherosclerotic lesion progression threefold compared to normal-salt controls. The pro-atherogenic effect was RAAS dependent, as it was attenuated by treatment with an angiotensin-converting enzyme inhibitor. More recently, McGraw et al. (13) demonstrated that low-dose Aldo infusion into ApoE^−/−^ mice increased atherosclerotic plaque burden and lipid content in the aortic root and arch after 4 weeks on a high-fat diet, compared to vehicle-infused controls. Aldo increased atherogenesis without a change in blood pressure. Aldo mainly promoted early atherosclerosis, as at 8 weeks the plaque size and lipid content in the aortic root had equalized between the two groups. Conversely, treatment with the MR antagonist eplerenone attenuated early (14), but not advanced, plaque development in ApoE^−/−^ mice (15, 16). In short, abundant data support a blood pressure-independent contribution of MR and Aldo to the pathogenesis of atherosclerosis in human and in animal models. This review summarizes recent advances in our understanding of the mechanisms by which the MR contributes to atherosclerosis by participating in initial endothelial injury, vascular inflammation, dysfunction of inflammatory cells, and the development of unstable plaques predisposed to thrombosis.

## MR in the Vascular Endothelium Contributes to Endothelial Cell Damage and Dysfunction Caused by Cardiovascular Risk Factors

**Figure 1:**
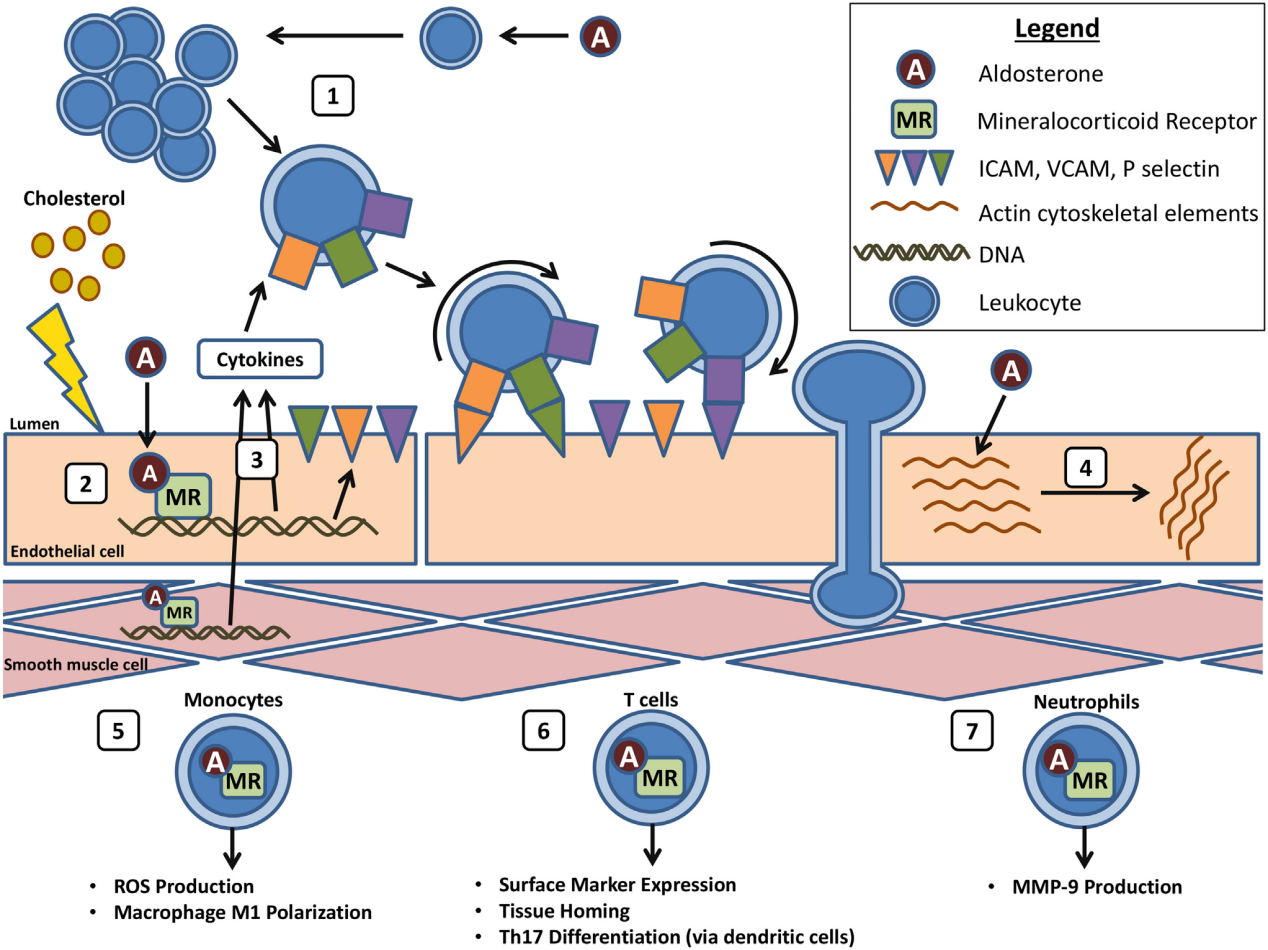
**Aldo and MR in vascular inflammation**. (1) Aldo induces systemic inflammation in the setting of cardiovascular risk factors. (2) In response to endothelial damage, Aldo acts on EC–MR to induce cytokine and adhesion molecule expression. (3) Cytokines produced by the endothelium and underlying SMCs induce EC adhesion molecule expression and leukocyte recruitment, adhesion, and transmigration. (4) Aldo also acts on EC–MR to induce rearrangement of the actin cytoskeleton, possibly facilitating leukocyte transmigration. (5) Myeloid MR induces ROS production and macrophage activation and polarization to the M1 phenotype. (6) MR induces T cell expression of homing markers and differentiation to the Th17 subset. (7) Aldo activation of neutrophil MR induces MMP-9 production, which may promote atherosclerotic plaque rupture.

Atherosclerosis begins with dysfunction of the vascular endothelium. Healthy endothelial cells (ECs) contribute to vasodilation by producing nitric oxide via endothelial nitric oxide synthase (eNOS) activity. Nitric oxide production is impaired early in atherogenesis, and the downstream effects of this can be measured *in vivo* by tonometry and flow-mediated vasodilation or *ex vivo* by quantifying achetylcholine-induced vasodilation of arterial rings. Aldo and vascular MR have been recently implicated in the development of endothelial dysfunction in humans with cardiovascular risk factors and in corresponding animal models. Hypertensive African-Americans had impaired endothelial function, as measured by pulse arterial tonography and by *ex vivo* studies of adipose vessels, which was improved with spironolactone treatment. Conversely, normotensive subjects had impaired endothelial function after Aldo administration, which was also prevented by spironolactone (17). Hypertensive subjects had an associated decrease in arteriolar glucose-6-phosphate dehydrogenase (G6PD) activity compared to non-hypertensives. A potential mechanism is suggested by a prior study demonstrating that Aldo treatment of bovine ECs *in vitro* and of mice *in vivo* decreases EC G6PD expression, resulting in excessive production of reactive oxygen species [ROS (18)]. In another study, treatment of diabetics with spironolactone improved coronary flow reserve, as measured by cardiac PET scan, compared to subjects treated with hydrochlorothiazide to achieve the same blood pressure (19). Thus, in patients with risk factors including hypertension and diabetes, endothelial dysfunction appears to be MR dependent (Figure 1).

Studies in animal models also support that the role of the MR in endothelial function depends on the health and integrity of the endothelium. Heylen et al. (20) found that arterioles from healthy male Wistar rats dilate in response to Aldo placed in the buffer bath, with even greater vasodilation when Aldo was administered intraluminally to directly target the endothelium. However, when the endothelium was denuded, Aldo instead induced vasoconstriction. This suggests a vasodilatory response in normal, healthy endothelium, and a vasoconstriction response (likely mediated by SMC–MR) when the endothelium is damaged or removed. To specifically interrogate the role of endothelial MR in vasodilatory function, several groups have used transgenic animal models with MR specifically deleted from ECs. Most studies demonstrated that EC–MR deletion does not alter endothelium-dependent relaxation in healthy aorta (21), mesentery, and coronary vascular beds (22) as measured using myographic techniques [although one study demonstrated decreased vasodilation in the aorta from EC–MR knockout mice (23)]. However, in animal models of cardiovascular risk factors, Aldo and the MR (likely in ECs) appear to contribute to the development of endothelial dysfunction. In a diet-induced obesity model, impaired endothelial-dependent vasodilation is prevented by MR inhibition with eplerenone or deletion of EC–MR (21). Similarly, endothelial dysfunction in a rat streptozotocin-induced diabetes model is MR dependent, as it was inhibited by spironolactone (24). In the stroke-prone spontaneously hypertensive rat model, spironolactone significantly improved endothelial function in the middle cerebral artery and reduced post-ischemia stroke infarct size (25). Finally, EC–MR knockout mice had improved endothelial function after 2 weeks of angiotensin-II-induced hypertension compared to MR-intact mice (22). In the same study, EC–MR was also found to contribute to coronary vasoconstriction in response to endothelin-1 and thromboxane in the presence and absence of hypertension. Taken together, these data support the concept that in healthy endothelium, Aldo induces vasodilation, whereas in the setting of cardiovascular risk factors, including obesity, diabetes, and hypertension, MR in the endothelium contributes to endothelial damage and the resulting impairment of endothelial-dependent vasodilation.

## Vascular MR and Aldo in the Development of Vascular Inflammation

### MR contributes to systemic inflammation in response to cardiovascular risk factors

Cardiovascular risk factors are associated with increased vascular inflammation, and Aldo and the MR have recently been implicated in this process. In patients with untreated essential hypertension, higher Aldo levels were associated with increased serum inflammatory and pro-thrombotic markers (26). Similarly, in obese young adults, high Aldo levels correlated with increased serum inflammatory markers and increased aortic stiffness, a risk factor for ischemic events (27). In the ApoE^−/−^ mouse atherosclerosis model, Aldo treatment increased circulating cytokines RANTES and MCP-1 and increased spleen size (13), and in a rat model of peritoneal dialysis, spironolactone treatment attenuated peritoneal inflammation and fibrosis by decreasing expression of MCP-1 and TGF-β (28). Overall, ample data support a link between MR activation and the development of inflammation in the setting of cardiovascular risk factors (Figure 1).

**Figure 2:**
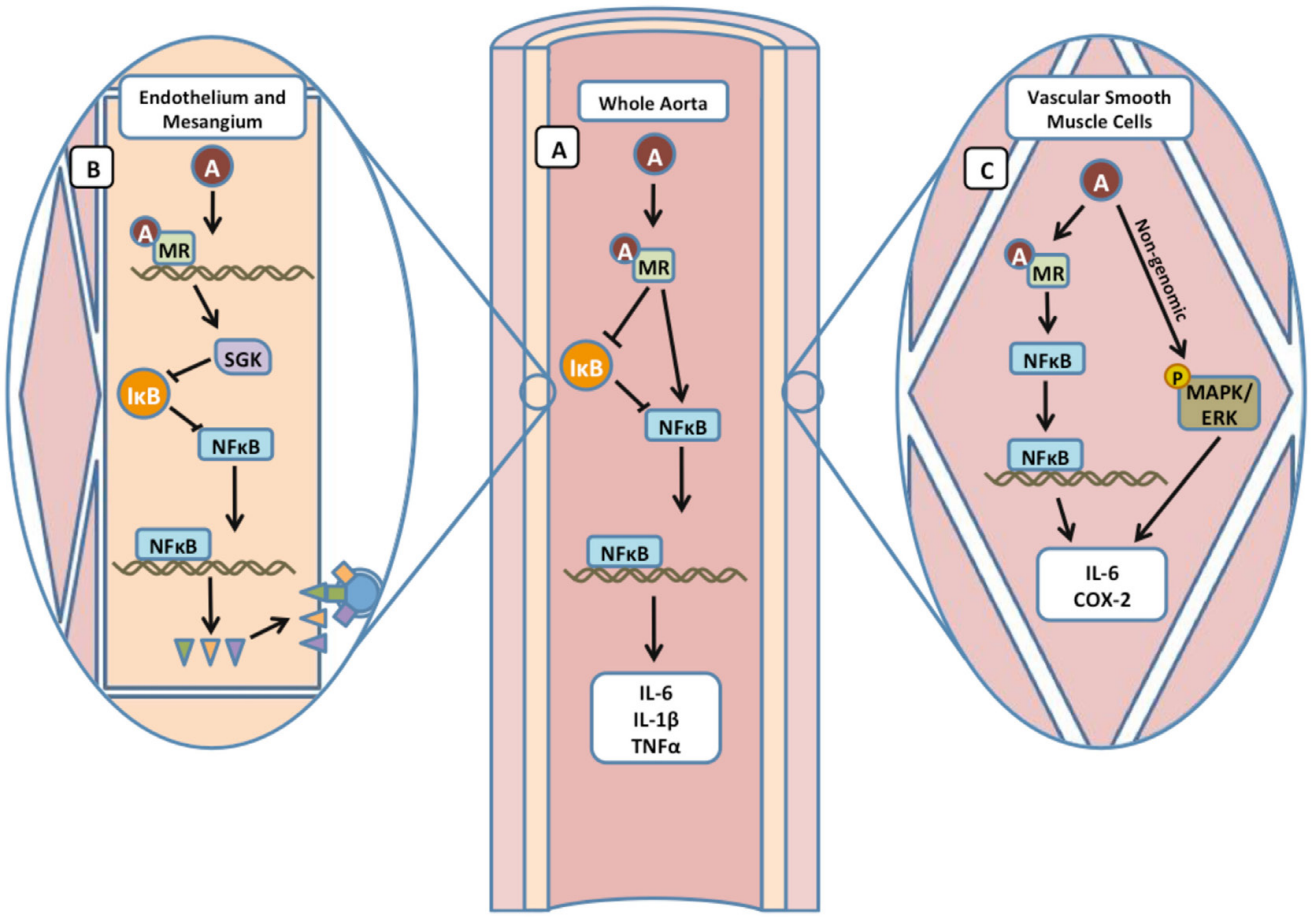
**Mineralocorticoid receptor contributes to vascular inflammation by activation of NFκB**. **(A)** In whole aorta samples, Aldo acting through the MR upregulates the expression of NFκB precursor subunit p105 and downregulates the NFκB inhibitor IκB, promoting transcription of cytokines IL-6, IL-1β, and TNFα. **(B)** In ECs, inhibition of the MR with eplerenone attenuates expression of NFκB targets ICAM-1, VCAM, and P-selectin (triangles). In rat mesangial cells, regulation of NFκB by the MR was mediated by SGK-1 inhibition of IκB. **(C)** Aldo exerts both genomic and non-genomic effects on NFκB target genes in SMCs.

One mechanism by which Aldo and the MR promote inflammation is by activation of the nuclear factor (NF)κB transcription factor, a critical regulator of cytokine and inflammatory gene expression [reviewed in Ref. (29)]. In primary cultured rat renal collecting duct cells, Aldo-induced NFκB activity and transcription of pro-inflammatory cytokines by a mechanism that required the classical MR genomic target, serum and glucocorticoid regulated kinase-1 [SGK-1 (30)]. In the vasculature of spontaneously hypertensive rats, expression of inflammatory markers IL-1β and IL-6 and the NFκB subunit p105 were increased and the NFκB inhibitor IκB was decreased compared to normotensive rats. The upregulation of NFκB and inflammatory markers was reversed by eplerenone but not by triple antihypertensive therapy, suggesting that the inflammation was due to MR activity rather than simply hypertension (31). Additionally, Aldo treatment of rat vascular SMCs induced NFκB translocation to the nucleus resulting in dose-dependent increases in cyclooxygenase-2 (COX-2) protein abundance and IL-6 mRNA transcripts. IL-6 and COX-2 levels decreased with the addition of inhibitors of the MR or of NFκB, confirming that SMC–MR activation by Aldo promotes inflammatory gene expression by NFκB activation. Aldo may also contribute to SMC inflammatory gene expression through a non-genomic mechanism involving activation of the MAP-kinase/ERK pathway (32). Altogether, these studies demonstrate a role for Aldo and the MR in the development of systemic and vascular inflammation through the downstream actions of NFκB (Figure 2).

### MR contributes to vascular leukocyte adhesion and trans-endothelial migration

When damaged, ECs produce cytokines and express adhesion molecules on their surface to attract circulating leukocytes. Specifically, activated ECs express P- and E-selectin, intracellular adhesion molecule (ICAM)-1, and vascular cell adhesion molecule (VCAM)-1, which interact with leukocyte surface proteins to promote leukocyte–endothelial adhesion and facilitate trans-endothelial migration of leukocytes into the vasculature [reviewed in Ref. (2)]. Aldo and the MR have been found to contribute to this process through effects both in the vasculature and in immune cells. In ApoE^−/−^ mice, Aldo treatment enhanced aortic leukocyte infiltration (13). In the same study, conditioned media from Aldo-treated SMCs promoted monocyte chemotaxis *in vitro*, suggesting a role for SMC–MR in leukocyte recruitment to atherosclerotic lesions. In primary human coronary ECs, MR activation increased ICAM-1 gene transcription, resulting in increased ICAM-1 surface protein, and ICAM-1-dependent leukocyte adhesion to human coronary ECs (33). Recently, estrogen acting through the estrogen receptor was found to inhibit MR induction of ICAM-1 expression in ECs and the associated leukocyte adhesion, providing a potential mechanism for sex differences in atherosclerosis in pre-menopausal women (34). ICAM-1 upregulation by MR in rat mesangial cells was found to be mediated by SGK-1-induced phosphorylation and inactivation of the NFκB inhibitor IκB, thereby allowing NFκB translocation to the nucleus to regulate ICAM-1 transcription (35). Kirsch et al. (36) later showed that treatment of human umbilical vein ECs with Aldo caused a rearrangement of F-actin cytoskeletal fibers and a subsequent increase in trans-endothelial permeability to labeled albumin in an MR- and MAPK/ERK-dependent manner. This effect of the MR on endothelial permeability could represent a mechanism by which the MR contributes to immune cell migration into the sub-endothelial space early in atherogenesis. Overall, these data support that MR activation in human SMCs and ECs may directly contribute to vascular inflammation by enhancing immune cell recruitment, adhesion, and trans-endothelial extravasation (Figure 1).

### MR effects on inflammatory cell function in atherosclerosis

In addition to direct effects on the vasculature, Aldo and MR may directly modulate leukocyte function [reviewed in Ref. (37)] to contribute to inflammation and atherosclerosis. In ApoE^−/−^ mice, eplerenone treatment reduced plaque size, macrophage infiltration, and oxidative stress (38). Moreover, peritoneal macrophages from eplerenone-treated ApoE^−/−^ mice had reduced capacity to oxidize low-density lipoprotein and to release superoxide ion (14), suggesting that MR may directly modulate macrophage function within the plaque. Indeed, macrophages from mice with MR genetically deleted from myeloid cells showed changes in gene expression profiles consistent with the non-classical M2 phenotype. These mice were also protected from hypertension-induced cardiac fibrosis and hypertrophy (39). *In vitro*, MR activation in macrophages promoted the M1 phenotype and MR deletion promoted M2 predominance (39, 40).

T-cell function also plays a role in atherosclerotic plaque inflammation and may be modulated by the MR. MR activation via fludrocortisone administration to healthy human subjects reduced the numbers of circulating naïve CD4^+^ and CD8^+^ T-cells and increased T-cell expression of CXCR4, a bone marrow-homing receptor, and CD26L and CCR7, proteins involved in migration to lymph nodes (41). Induction of hypertension in rats by deoxycorticosterone acetate and salt to activate the MR resulted in an increase in the Th17 subset of T-cells as evidenced by increased IL-17 and decreased forkhead box P3 expression in peripheral blood, heart, and kidney. This effect was reversed by treatment with spironolactone, suggesting a role for the MR in Th17 cell activation in the setting of hypertension (42), although this may be through MR action in dendritic cells rather than in T-cells (43).

Finally, the MR may also modulate neutrophil function, with potential effects on plaque stability. Treatment of human neutrophils with Aldo induced a dose-dependent increase in MMP-9 mRNA, which was attenuated by addition of spironolactone or of PI3K and ERK pathway inhibitors, implicating the MR and PI3K/ERK signaling in Aldo induction of neutrophil MMP-9 expression (44). Thus, Aldo and MR may also contribute to the pathogenesis and adverse outcomes of atherosclerosis by direct effects on leukocytes to promote macrophage ROS production and M1 polarization, modulate T-cell surface marker expression and Th17 differentiation, and increase neutrophil matrix protease expression (Figure 1).

## MR and Aldo in Plaque Rupture and Thrombus Formation

Although stable atherosclerotic plaques can cause symptoms, such as angina or claudication, morbidity and mortality is predominantly due to plaque rupture causing acute vessel occlusion, tissue ischemia and necrosis, resulting in MI, stroke, limb ischemia, and cardiovascular death. Plaque rupture occurs when the plaque fibrous cap is sufficiently degraded by MMPs to fracture and expose the thrombogenic core, resulting in activation of the clotting cascade. The role of the MR in the rupture and thrombosis of atherosclerotic plaques is poorly studied, partly due to a lack of rodent models of plaque rupture. MR activation has been shown to produce plaques with increased lipids and inflammatory cells, a phenotype more prone to rupture (13, 45), and as described above, the MR in neutrophils may contribute to MMP expression that destabilizes plaques. There is also some evidence that Aldo and the MR contribute to thrombosis. Aldo increased the rate of carotid artery thrombosis after thermal injury in the LDL-receptor-KO mouse model of atherosclerosis (46). In a rat model of thrombosis by vena cava ligation, acute Aldo administration enhanced thrombus burden, an effect that was prevented by co-treatment with eplerenone (47). Eplerenone also reduced post-carotid injury thrombosis in streptozotocin-induced diabetic rats (48). Aldo treatment has been shown to decrease bleeding time (49), induce platelet activation and degranulation, and increase expression of the thrombogenic plasminogen activator inhibitor [PAI-1 (50)], whereas MR inhibitors increase bleeding time (49), decrease platelet activation (50, 51), and inhibit expression of PAI-1, fibrinogen, P-selectin, and IL-1β in a variety of rodent models (48, 51, 52). Paradoxically, Ducros et al. (53) treated cultured human bone marrow ECs with Aldo and found an increase in the synthesis of the anti-thrombogenic endothelial protein C receptor (EPCR) and an increase in the clotting time of isolated plasma exposed to these ECs. Lagrange et al. (54) similarly found that a mouse model overexpressing human MR in ECs had increased EPCR expression and a decrease in thrombin at the EC surface *in vitro*. It is possible that rather than the traditional anti-thrombogenic protein C pathway, these latter findings could represent activation of the alternate cytoprotective pathway, which includes anti-inflammatory, anti-apoptotic, and gene expression effects [reviewed in Ref. (55)] and thus may represent a compensatory mechanism for adverse effects of global Aldo treatment, rather than an anti-thrombotic effect of Aldo. Overall, although MR appears to play some role in vascular thrombogenesis, additional studies are needed to clarify the molecular mechanisms, and whether this is relevant to thrombosis in the setting of atherosclerotic plaque rupture remains to be determined.

## Summary and Future Directions

In summary, recent data support that the MR plays a role in every step in the development and complications of atherosclerosis, including (1) development of endothelial dysfunction and systemic inflammation in response to cardiovascular risk factors; (2) EC and SMC production of cytokines and surface expression of leukocyte adhesion molecules, resulting in recruitment, adhesion, and transmigration of leukocytes from the circulation to the vasculature; (3) leukocyte activation, oxidative stress, and MMP expression; (4) development of plaque instability; and (5) vascular thrombosis (Figure 1). The many roles of MR in the development of atherosclerosis suggest potential novel therapies, including systemic MR inhibition, with currently available drugs or targeting of pathways downstream of vascular and leukocyte MR to prevent atherosclerosis complications, such as MI and stroke. The MR promotes vascular inflammation through the NFκB pathway in ECs and SMCs, although other pathways, including MAPK/ERK, are involved (Figure 2). Further exploration of the role of vascular and leukocyte MR in vascular inflammation and leukocyte function are needed to identify novel therapeutic targets downstream of MR activation. Finally, the interplay between the many cell types involved in atherosclerotic lesion development and its complications and the role of the MR in this interaction is an important area for future study.

## Conflict of Interest Statement

The authors declare that the research was conducted in the absence of any commercial or financial relationships that could be construed as a potential conflict of interest.
